# Development of a novel in vitro micronucleus test using human induced pluripotent stem cell-derived T lymphocytes

**DOI:** 10.1186/s41021-025-00345-9

**Published:** 2025-12-02

**Authors:** Ryota Kobayashi, Katsunori Sasaki, Ryoko Matsuyama, Koichi Saito, Ayako Kumagai, Shuichi Kitayama, Yohei Kawai, Shin Kaneko

**Affiliations:** 1 Environmental Health Science Laboratory, Sumitomo Chemical Co., Ltd, Osaka, 554-8558 Japan; 2 Center for iPS Cell Research and Application (CiRA), Kyoto University, 53 Kawahara-cho, Shogoin, Sakyo-ku, Kyoto, 606-8507 Japan

**Keywords:** Induced pluripotent stem cells, Human lymphocytes, In vitro micronucleus test, Genotoxicity, Toxicity

## Abstract

**Background:**

Various immortalized cells and human fresh blood lymphocytes have been used in in vitro genotoxicity studies (e.g., micronucleus (MN) test). Although immortalized cells can be supplied stably, their properties are different from normal cells such as abnormal karyotype. Human fresh blood lymphocytes are representative human normal cells, but homogenous lymphocytes are difficult to supply stably and in a timely manner due to individual differences between donors. Here, we aimed to develop a novel in vitro MN test using human induced pluripotent stem cell (hiPSC)-derived T lymphocytes to overcome the above problems.

**Results:**

hiPSCs were differentiated to T lymphocytes, which were confirmed to possess the ability to grow well in culture, a normal karyotype, and a spontaneous frequency of micronuclei. The genotoxicity of several reference positive / negative control substances was evaluated. The responses for all test substances, including clastogen, aneugen and negative substances, were consistent with published reports.

**Conclusions:**

Our results demonstrated promising proof-of-principle data as an in vitro MN test and suggest that hiPSC-derived T lymphocytes have a potential to make a significant contribution to the improvement of in vitro genotoxicity studies.

## Introduction

 To evaluate chromosomal aberrations, in vitro chromosomal aberration tests, in vitro micronucleus (MN) tests, and in vitro comet assays that detect DNA damage related to the chromosome are widely used. While various cell types can be used to evaluate chromosomal aberrations in vitro, mammalian-derived immortalized cells, such as the Chinese hamster lung-derived fibroblast cell lines CHL/IU [1] and V79 [2] and the Chinese hamster ovary-derived cell line CHO [3], are widely used because they are easy to handle and show high sensitivity. The human lymphoblastoid cell line TK6 [4], which is a human immortalized cell line, and human lymphocyte (HuLy) derived from fresh blood are also used [5]. However immortalized cells derived from mammals show false positive results [6, 7]. The cause may be the dysfunction of the tumor-suppressor gene cell cycle regulator *p53* and abnormalities in the karyotype [8]. While TK6 has normal p53 function, it has several chromosomal aberrations, including trisomy of chromosome 13, translocation of chromosome 14 and chromosome 20, and translocation of chromosome 3 and chromosome 21. In addition, one report suggested that factors other than p53 function in immortalized cells also contribute to false positive results in in vitro genotoxicity tests [9].

Accordingly, attention is on normal human cells. HuLy requires the collection of fresh human blood for every test since its proliferation ability is limited. Therefore, it is difficult to ensure a stable supply of cells, since donors must meet various criteria such as age, medical history, smoking history, and more. Plus, HuLy is known to show slightly different properties between donors, resulting in different test results. In addition, the cell profile depends on the donor’s physiological condition at the time of collection. Finally, the number of cells derived from fresh blood is limited.

Human embryonic stem cells (hESCs) and human induced pluripotent stem cells (hiPSCs) are alternative cell lines that produce differentiated cells of high quality and homogeneity. Indeed, we have proposed in vitro phototoxicity assays using retinal pigment epithelial cells derived from hESCs [10]. Nishimura et al. [11] developed a protocol to induce hiPSCs into functionally T lymphocytes that have a high proliferative capacity and elongated telomeres and are applicable to adoptive immunotherapy. In this study, we developed an in vitro MN test using hiPSC-derived T lymphocytes.

## Material and methods

### Test substances

All 13 test substances used have sufficiently been evaluated previously [3, 5, 9, 12–19] and are summarized in Table 1. Mitomycin C (MMC) was purchased from Kyowa Hakko Kirin Co., Ltd. (Tokyo, Japan); cytosine arabinoside (AraC), *N*-methyl-*N*-nitro-*N*-nitrosoguanidine (MNNG) and Cyclophosphamide monohydrate (CPA) from Nacalai Tesque Inc. (Kyoto, Japan); *N*-ethyl-*N*-nitrosourea (ENU), benzo[a]pyrene (B[a]P) and methyl methanesulfonate (MMS) from Sigma-Aldrich (St. Louis, MO, U.S.A.); and 4-nitroquinoline 1-oxide (4NQO), etoposide (ETO), colchicine (COL), vinblastine sulfate (VBL), D (-)-mannitol (MAN), and sodium chloride (NaCl) from Wako Pure Chemical Industries, Ltd. (Osaka, Japan).

For exposure, the test substances were dissolved in appropriate solvents, e.g., dimethyl sulfoxide (DMSO), physiological saline, or in culture medium just before use. The final concentration of the solvents (DMSO or physiological saline) was 1.0% (v/v) in culture medium.

**Table 1:** List of test substances

Category	Test substances	CAS No.	Solvent
Clastogen active without metabolic activation			
	MMC	50-07-7	physiological saline
	AraC	147-94-4	DMSO
	ENU	759-73-9	DMSO
	MMS	66-27-3	DMSO
	MNNG	70-25-7	DMSO
	4NQO	56-57-5	DMSO
	ETO	33419-42-0	DMSO
Clastogen active only with metabolic activation			
	CPA	6055-19-2	physiological saline
	B[a]P	50-32-8	DMSO
Aneugen			
	COL	64-86-8	physiological saline
	VBL	143-67-9	DMSO
Negative substance			
	MAN	69-65-8	physiological saline
	NaCl	7647-14-5	culture medium

### Generation of hiPSC-derived T lymphocytes

hiPSCs were established from cryopreserved human peripheral blood mononuclear cells (Cellular Technology Limited and Precision For Medicine, U.S.A.) and differentiated into T lymphocytes at the Center for iPS Cell Research and Application, Kyoto University, as previously described [11]. Frozen stocks of lymphocytes were prepared and used for each experiment.

### Sequence analysis of *p53* cDNA and karyotype analysis of HiPSCs

Total RNA was extracted from hiPSCs using an RNeasy mini kit (Qiagen, Hilden, Germany), and cDNA was prepared using SuperScript III Reverse Transcriptase (Invitrogen, California, U.S.A.). Using KOD -Plus- Neo (Toyobo Co., Ltd., Osaka, Japan) and *p53*-specific PCR primers (5’-ATGGAGGAGCCGCAGTCAGATC-3’ and 5’-TCAGTCTGAGTCAGGCCCTTC-3’), PCR was performed. The PCR product was subcloned into the vector pTA2 (Toyobo Co., Ltd.) using TArget Clone -Plus- (Toyobo Co., Ltd.), and the DNA was extracted. Using a 3730XL Genetic Analyzer (Applied Biosystems, California, U.S.A.) and the primers 5’-GTAATACGACTCACTATAGGGC − 3’, 5’-TGATTTGATGCTGTCCCCGGAC − 3’, and 5’-ACAGCACATGACGGAGGTTGTG-3’, *p53* cDNA was analyzed. The sequences were compared with TK6-derived sequences that have been reported to be normal [20]. The karyotype of hiPSCs was determined by LSI Medience Corporation (Tokyo, Japan) using the standard staining protocol for Giemsa-banding [21].

### Flow cytometry analysis of hiPSC-derived T lymphocytes

Cells were incubated with the appropriate concentration of antibodies: anti-CD3 antibody (Clone: HIT3a, Isotype: Mouse IgG2a, Biolegend, California, U.S.A.) and anti-TCRαβ antibody (Clone: WT31, Isotype: Mouse IgG1, eBioscience, California, U.S.A.) for 30 min at 4℃ and washed with Dulbecco’s phosphate buffered saline without calcium chloride or magnesium chloride (DPBS (-)). Propidium iodide (Dojindo Lab., Kumamoto, Japan) was added to exclude dead cells. Stained cell samples were analyzed using a flow cytometer (MA900, SONY, Tokyo, Japan) and selected and analyzed using the gates set by cryopreserved human peripheral blood mononuclear cells.

### Proliferative stimulation

For the proliferation stimulation of hiPSC-derived T lymphocytes, an anti-CD3 antibody (Clone: OKT3, Isotype: Mouse IgG2a, eBioscience)–coated cell culture plate was used. The cells were suspended and seeded at a density of 1.0–2.0 × 10^6^ cells/mL on the antibody-coated plate and incubated at 37℃ in 5% CO_2_. After 17 h of incubation, the cell suspension was diluted to a cell density of 2.0–4.0 × 10^5^ cells/mL and reseeded in a plate not coated with the antibody. The culture was continued for 21 h.

### Exposure

The highest concentration used for each test substance corresponds to 10 mM or 2 mg/mL, whichever is the lowest. Furthermore, the highest concentration for each positive control substance was adjusted by its cytotoxicity based on our earlier studies.

For the test substances that did not need metabolic activation, cytochalasin B (CytoB; Wako Pure Chemical Industries, Ltd.) at a final concentration of 6 µg/mL was added with the test substances to the cell suspension, which was then incubated for about 28 h (long-term treatment).

Phenobarbital and 5,6-benzoflavone-induced rat liver S9 (Oriental Yeast Co., Ltd., Tokyo, Japan) was used to metabolically activate CPA and B[a]P. S9 mix (glucose-6-phosphate, 5 mM; NADPH, 4 mM; MgCl_2_, 5 mM; KCl, 33 mM; HEPES buffer, pH 7.2, 4 mM; and S9 fraction, 30%) was prepared just before use. S9 mix (final concentration, 1.0% (v/v)) and the test substances were added to the cell suspension, which was then incubated for about 3 h (short-term treatment). Afterwards, the cell suspension was washed with DPBS (-) three times, then culture medium containing CytoB was added, and the culture was continued for about 25 h. All the above exposures at each concentration were performed in single cell culture.

### Preparation of slides and staining

The cell suspension after treatment and completion of the culture was hypotonized in 75 mM KCl and fixed in solution (methanol : glacial acetic acid = 3 : 1, and methanol : glacial acetic acid = 98 : 2). The cells were suspended in a small amount of 2% fixation solution at a cell density at which the suspension became slightly cloudy, and the suspension was added dropwise on a slide glass and air dried for MN observation. The specimen was stained with 40 µg/mL Acridine Orange (Wako Pure Chemical Industries, Ltd.), and the cells were observed under a fluorescence microscope (BX51 System Microscope; Olympus, Tokyo, Japan) using a wide-band Blue excitation filter.

### Micronucleus analysis

All slides were coded and analyzed in a blinded manner. At least 500 cells were scored per slide to determine the percentage of mononucleate, binucleate, and multinucleate cells. In addition, at least 1,000 total binucleate cells were observed per slide to determine the percentage of micronucleated binucleate cells. For conditions under which 500 cells or binucleated cells could not be observed due to cytotoxicity of the test substance, all cells or binucleated cells on a slide glass were observed. The frequency of micronucleated binucleate cells was analyzed using Fisher’s exact test (two-sided, significant level of 0.05) with the negative control group.

As an index of cytotoxicity, RI (replication index) was calculated according to the OECD TG 487 [22] as follows:

$$\frac{\begin{gathered} (\left( {1{\text{ }} \times {\text{ }}Number{\text{ }}of{\text{ }}binucleated{\text{ }}cells} \right) + {\text{ }} \hfill \\ \left( {2{\text{ }} \times {\text{ }}Number{\text{ }}of{\text{ }}multinucleate{\text{ }}cells} \right))/ \hfill \\ Total{\text{ }}number{\text{ }}of{\text{ }}cells{\text{ }}treated{\text{ }}cultures \hfill \\ \end{gathered} }{\begin{gathered} (\left( {1{\text{ }} \times {\text{ }}Number{\text{ }}of{\text{ }}binucleated{\text{ }}cells} \right) + {\text{ }} \hfill \\ \left( {2{\text{ }} \times {\text{ }}Number{\text{ }}of{\text{ }}multinucleate{\text{ }}cells} \right))/ \hfill \\ Total{\text{ }}number{\text{ }}of{\text{ }}cells{\text{ }}control{\text{ }}cultures \hfill \\ \end{gathered} } \times 100$$

## Results

### Generation of hiPSC-derived T lymphocytes

**Fig. 1:**
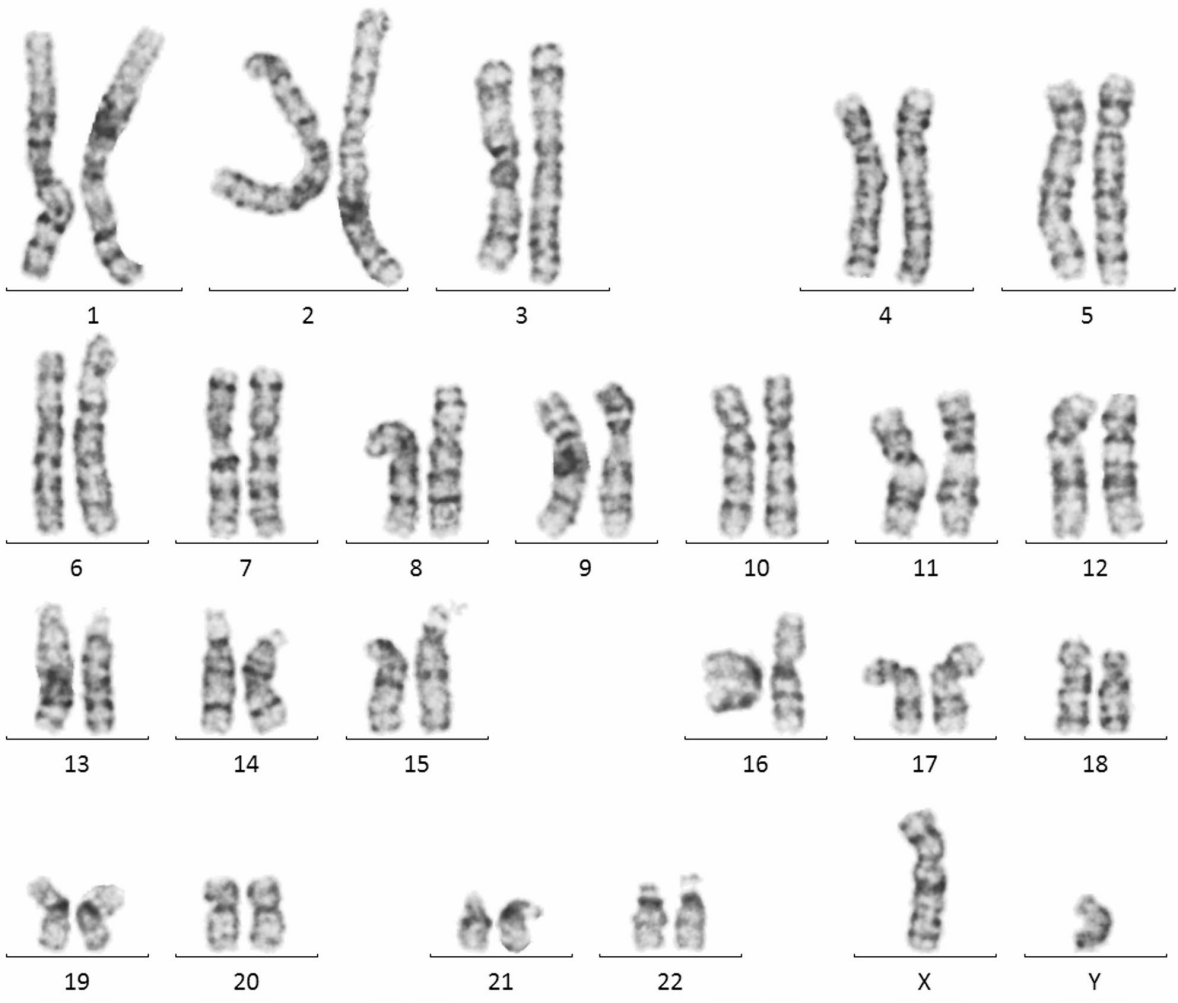
Karyotype analysis of the selected hiPSC clone (F1 R(-) #2) using Giemsa staining

**Fig. 2:**
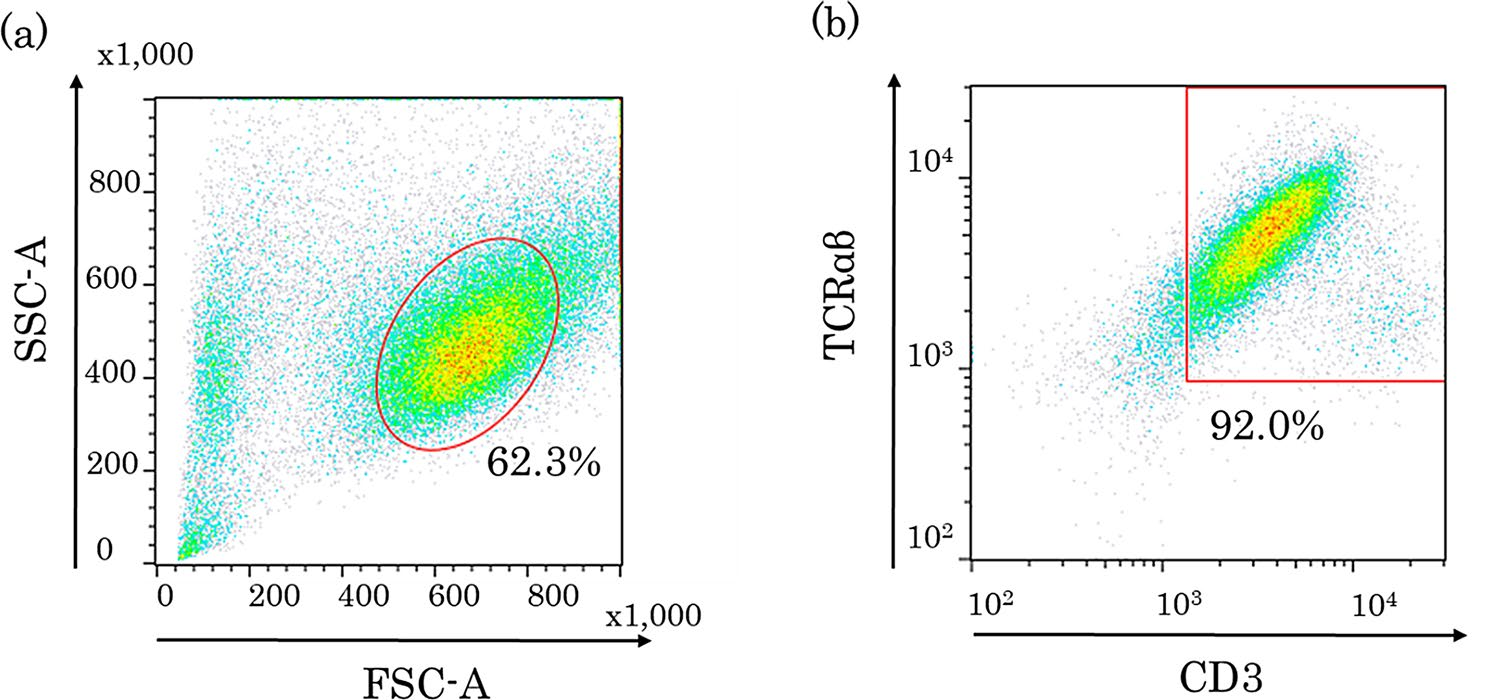
Flow cytometry analysis of hiPSC-derived T lymphocytes. (**a**) Complexity (SSC-A) versus cell size (FSC-A) with a gate that distinguish cells from debris, etc. (**b**) Co-expression of TCRαβ and CD3 in the gated population

Before generating hiPSC-derived T lymphocytes, the spontaneous frequencies of MN in five hiPSC clones were investigated. A chi-square test showed a statistically significant difference in the spontaneous frequencies of MN among five iPSC clones (*p* < 0.01) (Table 2). When some hiPSC clones were differentiated, no remarkable change in the trend in the spontaneous frequency of MN before and after differentiation was observed (data not shown). Therefore, an hiPSC clone with a low spontaneous frequency of MN was selected. The selected clone showed no abnormal karyotype but normal diploidy (2n = 46, Fig. 1), and the *p53* gene had no abnormality in its sequence (data not shown). Further, the cells expressed TCRαβ and CD3, which are both cell surface markers of T lymphocytes, at 92.0% (Fig. 2.). Collectively, these results suggest that hiPSC-derived T lymphocytes are reliable for detecting the induction of MN.

**Table 2:** Spontaneous frequency of micronuclei in hiPSCs

Clone number of the hiPSCs	% MN^a^
1	1.8
2	1.9
3	2.6
4	3.2
5	4.4

^a^ % MN = % micronucleated mononucleated cells = (total number of micronucleated mononucleated cells / total number of mononucleated cells analyzed (2,000 cells)) × 100. Micronucleated mononucleated cells were examined using the same procedure used for the in vitro MN test

### In vitro micronucleus test

**Fig. 3:**
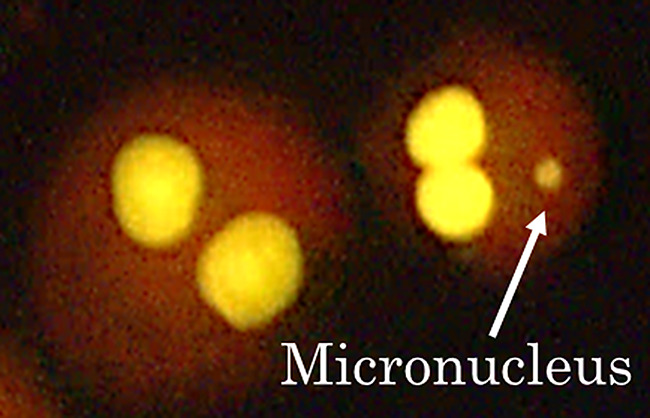
An example of a micronucleus in a binucleated cell. Cells were stained with Acridine Orange and photographed under UV-illumination

Before conducting the in vitro MN test, we confirmed that the solvents DMSO and physiological saline in culture medium (1.0% (v/v)) had no cytotoxicity, as estimated by the cytokinesis-block proliferation index (CBPI). In the solvent control group, CBPI values ranged from 1.5 to 1.8 indicating that cell proliferation occurred during CytoB-treated cultivation. The genotoxicity of clastogens that do not need metabolic activation (MMC, AraC, ENU, MMS, MNNG, 4NQO and ETO), aneugens (COL and VBL), and negative substances (MAN and NaCl) was evaluated in the long-term treatment condition without S9, and the genotoxicity of clastogens that need metabolic activation (CPA and B[a]P) was evaluated in the short-term treatment condition with S9. An example of MN in a binucleated cell is shown in Fig. 3. The results of the in vitro MN test are shown in Table 3. Concentrations that caused excessive toxicity (RI < 40%) were excluded from the data analysis. For the clastogens and aneugens, dose-dependent and statistically significant increases in the MN frequency as compared to the concurrent solvent control were observed. In contrast, for all negative substances, no statistically significant increase in MN frequency as compared to the concurrent solvent control was observed up to the maximum dose (10 mM). Overall, the positive /negative responses obtained for all test substances are consistent with published reports [3, 5, 9, 12–19].

**Table 3:** In vitro MN test results

Test substances	Concentration (mM)	% Toxicity (RI)	% MN^a^	Statistical significance (Fisher’s)
Clastogen active without metabolic activation				
MMC				
	0	100	1.10	-
	0.000075	90.1	1.40	N/S
	0.00015	55.8	1.80	N/S
	0.0003	65.3	2.60	*p < 0.05
	0.00045	51.2	2.60	*p < 0.05
AraC				
	0	100	0.90	-
	0.000041	63.1	0.95	*p < 0.05
	0.000082	56.7	1.65	*p < 0.05
	0.00012	52.1	1.70	*p < 0.05
ENU				
	0	100	1.40	-
	0.21	80.6	2.90	*p < 0.05
	0.43	65.9	5.70	**p < 0.01
MMS				
	0	100	2.10	-
	0.028	87.8	2.20	N/S
	0.057	90.2	4.10	*p < 0.05
	0.11	72.5	5.61	**p < 0.01
MNNG				
	0	100	1.40	-
	0.00085	83.9	2.80	*p < 0.05
	0.0017	69.2	5.10	**p < 0.01
	0.0034	56.0	7.96	**p < 0.01
4NQO				
	0	100	0.90	-
	0.000011	96.0	1.20	N/S
	0.000032	88.5	2.70	**p < 0.01
	0.000053	80.2	7.20	**p < 0.01
ETO				
	0	100	1.40	-
	0.00011	102	5.20	**p < 0.01
	0.00021	106	4.60	**p < 0.01
	0.00042	44.0	11.0	**p < 0.01
Clastogen active only with metabolic activation				
CPA				
	0	100	0.60	-
	0.0019	72.1	1.50	N/S
	0.0038	49.4	1.10	N/S
	0.0077	42.0	0.90	N/S
	0.011	44.6	2.80	**p < 0.01
	0.015	46.5	2.60	**p < 0.01
B[a]P				
	0	100	1.20	-
	0.05	45.1	0.90	N/S
	0.1	62.3	1.70	N/S
	0.2	55.6	1.90	N/S
	0.3	61.5	3.10	**p < 0.01
Aneugen				
COL				
	0	100	3.33	-
	0.0000075	93.9	4.28	N/S
	0.000013	78.8	5.44	*p < 0.05
	0.000018	57.6	9.84	**p < 0.01
	0.000023	57.6	7.17	**p < 0.01
VBL				
	0	100	3.33	-
	0.0028	57.2	8.44	**p < 0.01
	0.0055	45.0	5.47	N/S
Negative substance				
MAN				
	0	100	1.50	-
	2.5	98.8	0.80	N/S
	5	89.6	1.10	N/S
	10	76.1	1.00	N/S
NaCl				
	0	100	1.00	-
	2.5	99.1	0.80	N/S
	5	78.4	1.10	N/S
	10	89.7	1.40	N/S

N/S = not significant

^a^ % MN = % micronucleated binucleated cells = (total number of micronucleated binucleated cells / total number of binucleated cells analyzed) × 100

## Discussion

The immortalized cells generally used in in vitro MN tests have the advantage of being stably supplied. However, some of them have abnormalities in their karyotype and *p53* gene, which can affect the sensitivity of the tests. While HuLy are obtained from healthy donors, they suffer from lot-to-lot variability and supply issues. The hiPSC-derived T lymphocytes used in this study are capable of being frozen and thawed after expansion culture, allowing for a stable and homogeneous supply. Additionally, they are human cells with normal karyotype and *p53* gene and sufficiently express cell surface markers of T lymphocytes. These findings suggest that hiPSC-derived T lymphocytes have a potential to solve the issues burdening immortalized cells and Huly in in vitro MN tests.

The OECD TG 487 [22] states that cells used in in vitro MN tests are selected on the basis of their ability to grow well in culture, stability of their karyotype (including chromosome number), spontaneous frequency of MN, and observable induction of MN. To better understand the characteristics and assess the suitability of the cells used in genotoxicity studies, several types of properties are considered, such as cell division. We confirmed good cell division when anti-CD3 antibody was used as a proliferation stimulation factor under optimized culture conditions. Furthermore, it is better to use cells that have a lower spontaneous frequency of MN, as this property reflects more stable and sufficient sensitivity for the test and a normal cell type. The selected hiPSC clone and its differentiated T lymphocytes used in this study had low frequencies of MN. Although there have been no previous reports on the spontaneous frequency of MN in hiPSCs, we found that there were large variations in the spontaneous frequency among clones. Accordingly, we selected an hiPSC clone with a low spontaneous frequency, normal karyotype, and good differentiation efficiency. In the in vitro MN test, these cells responded to all test substances in a manner consistent with published reports [3, 5, 9, 12–19]. These results demonstrated promising proof-of-principle data as an in vitro MN test and has a potential to make a significant contribution to the improvement of current in vitro MN tests. The remarkable achievement indicated that Japan’s world-renowned induced pluripotent stem cell-related technology can also be applied to in vitro genotoxicity assessments. In the future, it is expected that this testing system will be authorized through additional verification, such as validation in compliance with OECD TG487 in multiple laboratories using more reference substances including different types of aneugens and a wide range of negative substances.

Many studies have shown that there are inter-individual differences in the susceptibility to genotoxic substances. Among the causes is aging, as one study showed genotoxin exposure led to an increase in chromatin instability in the spermatozoa of old animals [23]. Gender is another factor, as a synthetic estrogen was found to be more genotoxic in pre-pubertal mice, but males in particular [24]. Understanding such differences is important for identifying individuals who may be more susceptible to genotoxic substances and for developing strategies to prevent exposure and reduce risk. One of the human cell models for investigating inter-individual differences is HuLy, which can be collected from each individual. However, there are some problems with using HuLy. For example, lifestyle factors such as nutritional imbalance, lack of regular exercise, insufficient sleep, and overtime working contribute to higher spontaneous frequency of MN in HuLy [25]. Also, since the spontaneous frequency of MN in HuLy is very low, even slight variations in the frequency between experiments can affect the results. On the other hand, hiPSC-derived T lymphocytes can be obtained from almost any donor stably and homogeneously, thus enabling investigation of inter-individual differences in susceptibility to genotoxic substances more accurately.

## Conclusion

We developed a novel in vitro MN test using hiPSC-derived T lymphocytes.

## Data Availability

The datasets used during the current study are available upon reasonable request.
